# Correction: The Exponentiated Exponential Burr XII distribution: Theory and application to lifetime and simulated data

**DOI:** 10.1371/journal.pone.0268980

**Published:** 2022-05-19

**Authors:** The PLOS ONE Editors

## Notice of republication

At the time of retraction, the article [1] remained available online with an amended copyright statement. The retracted article was subsequently removed from the *PLOS ONE* website on May 11, 2022. The Retraction [2] was republished on May 11, 2022 to include an update notifying readers of the removal of the article content.
